# Hyaluronidase for Dermal Filler Complications: Review of Applications and Dosage Recommendations

**DOI:** 10.2196/50403

**Published:** 2024-01-17

**Authors:** George Kroumpouzos, Patrick Treacy

**Affiliations:** 1 GK Dermatology, PC South Weymouth, MA United States; 2 Department of Dermatology Warren Alpert Medical School at Brown University Providence, RI United States; 3 Ailesbury Clinics Ltd Dublin Ireland

**Keywords:** hyaluronidase, hyaluronic acid, filler, complications, nodule, vascular occlusion, therapy, treatment, application, dosage, management, skin, data, inflammatory nodule, inflammatory, injection

## Abstract

**Background:**

Hyaluronidase (Hyal) can reverse complications of hyaluronic acid (HA) fillers, which has contributed substantially to the popularity of such procedures. Still, there are differing opinions regarding Hyal treatment, including dosage recommendations in filler complication management.

**Objective:**

We aimed to address unanswered questions regarding Hyal treatment for HA filler complications, including timing and dosage, skin pretesting, properties of various Hyals and interactions with HA gels, and pitfalls of the treatment.

**Methods:**

PubMed and Google Scholar databases were searched from inception for articles on Hyal therapy for filler complications. Articles were evaluated regarding their contribution to the field. The extensive literature review includes international leaders’ suggestions and expert panels’ recommendations.

**Results:**

There are limited controlled data but increasing clinical experience with Hyal treatment. The currently used Hyals provide good results and have an acceptable safety profile. Nonemergent complications such as the Tyndall effect, noninflamed nodules, and allergic or hypersensitivity reactions should be treated with low or moderate Hyal doses. Hyal should be considered with prior or simultaneous oral antibiotic treatment in managing inflammatory nodules. Hyal may be tried for granulomas that have not responded to intralesional steroids. Emergent complications such as vascular occlusion and blindness require immediate, high-dose Hyal treatment. Regarding blindness, the injection technique, retrobulbar versus supraorbital, remains controversial. Ultrasound guidance can increase the efficacy of the above interventions.

**Conclusions:**

Hyal is essential in aesthetic practice because it can safely treat most HA filler complications. Immediate Hyal treatment is required for emergent complications. Aesthetic practitioners should be versed in using Hyal and effective dosage protocols.

## Introduction

Fillers are classified into three major classes based on their longevity in the tissues, which in turn depends on their structure and composition: (1) temporary, lasting less than 18 months; (2) semipermanent, lasting greater than 18 months; and (3) permanent, lasting longer than 24 months. It is generally believed that permanent fillers are nonbiodegradable and nonreversible, and therefore, complications with the inflammatory process are more likely to occur with permanent fillers. Dermal fillers have gained popularity over the past 2 decades despite the large spectrum of complications associated with their use, including nodule formation, misplacement, migration, infection, and vascular occlusion [1-3]. Hyaluronic acid (HA) fillers are temporary or semipermanent and remain the most used filler type [4]. Fillers that cannot be dissolved by hyaluronidase (Hyal), such as poly-l-lactic acid, calcium hydroxylapatite, and polymethylmethacrylate, are not discussed here. The ease and efficacy of Hyal in reversing HA gels’ (HAG’s) complications have contributed to such fillers’ popularity [5]. Performing Hyal injections under high-frequency ultrasound (HFUS) guidance, a recent advance in soft tissue augmentation, allows for higher accuracy and efficacy of the treatment, thereby maximizing the benefits [6].

Still, there has been a small number of well-designed randomized controlled trials (RCTs) on Hyal injections in aesthetics. Borzabadi-Farahani et al [7-11] found only 5 RCTS evaluating the effectiveness of Hyal in removing uncomplicated HA nodules. Dosing recommendations are often based on the suggestions of leading authorities and assessment by expert panels. The objective of this review is to discuss the aesthetic applications of Hyal injections and provide an updated assessment of dosing recommendations, including dosage (international units [iu]), treatment sessions, and incremental dose adjustments (titration). We discuss gaps and present our experience with Hyal treatments.

## Methods

We have completed a narrative review, as a systematic review is not feasible due to the high heterogeneity of articles on this broad topic. We searched PubMed and Google Scholar databases from inception for articles on Hyal therapy for filler complications. Complication is an adverse effect emphasizing direct causality between the filler procedure and the adverse outcome or event [1]. Key terms in the search included “complication OR adverse event,” “safety,” “prevention,” “management OR treatment OR intervention,” “hyaluronidase,” and “filler.” We performed separate searches for important complications using the terms “reaction,” “granuloma,” “nodule,” “infection OR biofilm,” “vascular occlusion OR vascular compromise,” and “skin necrosis.” A separate search for using ultrasound (key term “ultrasound”) in filler procedures was performed. We searched the reference lists of relevant articles. We included expert opinions, panel recommendations, and professional body guidance.

### Results and Discussion

#### Principal Findings

We review the findings of publications relevant to Hyal action [12-23], products available [14,18,19,21-23], reconstitution and storage [1,18,20,21,24,25], dosage considerations [5,9-11,16,18,21,26-30], skin pretesting [18,21,24,31-33], use in the management of filler complications [1,2,5-8,16,18,21,24,34-75], and the pitfalls of Hyal treatment [1,13,19,24,37,76-79].

#### Action of Hyal

Hyal is an endoglycosidase that can depolymerize HA leading to its degradation into monosaccharides by hydrolyzing the disaccharides at hexosaminidic β-1 through β-4 linkages [12]; however, it also breaks down to some extent other polysaccharides in the connective tissue [13,14]. In humans, 6 Hyals have been identified (HYAL-1, -2, -3, -4, HYALP1, and PH-20) [15]. Hyal has an immediate effect and a half-life of 2 minutes with the duration of action being 24 to 48 hours [16,17]. However, it is effective for a longer time period which may be related to the fact that a low number of iu is required to have a clinically significant effect; thus, even when the Hyal has mostly degraded, its action continues [18]. Commendably, Hyal breaks cross-links in the HA filler, which behaves like native HA in the skin, which has a half-life of 24 to 48 hours [15]. Hyal dissolves native HA, but the body restores native HA in 15-20 hours [19]; therefore, there are no detrimental long-term effects of Hyal on skin quality.

Hyal is a tissue permeability modifier and is indicated as an adjuvant in subcutaneous fluid administration for achieving hydration, increasing the dispersion and absorption of other injected drugs such as anesthetics and in subcutaneous urography for improving resorption of radiopaque agents [20]. Hyal is used off-label in in aesthetics.

#### Available Hyals

Hyals are derived from mammals (obtained from the testes), hookworms or leeches, and microbes [19]. Animal origin Hyals have been used clinically for almost 80 years [21]. Hyals that are currently available are of either animal origin or human recombinant (Table 1). Food and Drug Administration -approved Hyals include bovine (Amphadase), ovine (Vitrase) products, and recombinant human (Hylenex) products. Still, in many countries, only 1 Hyal type is available—Hylase “Desau” in Germany and “Hyalase” in the United Kingdom require reconstitution (product should be used within 6 hours) [14,22]. Recombinant human Hyal has a purity 100 times higher than some of the bovine preparations [23]. The recombinant type is thought to have a lower incidence of allergic reactions than animal-derived products that are more immunogenic, but long-term data are lacking [18,21].

**Table 1:** Some of the commercially available Hyal products^a^.

Trade name, country of origin	Source	Product details	Reconstitution required	Storage
Amphadase, United States^b^	Bovine	150 iu/mL in 2 mL vial; contains thimerosal	No	2 °C-8 °C
Hydase, United States^b^	Bovine	150 iu/mL in 2 mL vial	No	2 °C-8 °C
Hylenex, United States^b^	Human recombinant	150 iu/mL in 2 mL vial; contains human albumin	No	2 °C-8 °C
Vitrase, United States^b^	Ovine	200 iu/mL in 2 mL vial; contains lactose	No	2 °C-8 °C
Hylase “Desau,” Germany	Bovine	150, 300, 1500 iu/mL in vial	Yes	25 °C±2 °C; 60% relative humidity
Hyalase, United Kingdom	Not specified	1500 iu/mL in vial	Yes	≤25 °C

^a^Pregnancy category C.

^b^Food and Drug Administration–approved.

#### Reconstitution and Storage

Hyal is reconstituted in bacteriostatic normal saline, which is less painful upon injection than water and has additional anesthetic properties [18]. Bacteriostatic normal saline contains benzyl alcohol to prevent bacterial contamination. An aseptic technique should be used during the reconstitution process. One should gently swirl or mix the vial to dissolve the Hyal powder in the saline and avoid vigorous shaking to prevent foaming. The volume of the diluent depends on the indication and surface area to be treated, and a range of 1 to 10 mL has been evidenced in clinical practice [1]. Increased volumes of diluent or subsequent dilutions of a fraction of the reconstituted Hyal are needed if a small number of Hyal units are injected. These authors reconstitute 1500 iu Hyal (Hyalase) in 1 mL bacteriostatic saline and subsequently dilute fractions of the reconstituted Hyal product to achieve the desired number of units per 0.1 mL.

There is a theoretical concept that using a lower dilution (higher Hyal concentration) might provide a more focused effect, especially when targeting specific areas like nodules.

While a lower dilution may theoretically lead to more localized effects, it is crucial to balance this with the risk of excessive filler degradation by Hyal which can result in a complete loss of the aesthetic benefit of the filler procedure.

Some authors suggested diluting Hyal in lidocaine to decrease pain in cases of vascular occlusion [24]. However, this has not gained wide support as the enzymatic action of Hyal can be affected by pH and the pH of low lidocaine concentrations is not ideal for Hyal [18]. Additionally, there is a risk of widespread, increased systemic absorption of the anesthetic and potential complications. No evidence supports using lidocaine, with or without epinephrine, solvent to reduce bruising. In a report, a patient presented with soft blue nodules post-HA filler in bilateral infraorbital areas. The lesions were treated with 75 iu Hyal (reconstituted in 1 mL 1% lidocaine with epinephrine); lidocaine with epinephrine was selected to reduce bruising but was ineffective [25].

The Hyal products approved by the Food and Drug Administration (Table 1) should be stored at cool temperatures (2 °C-8 °C) to maintain the quality of the product over a long period of time [18,21]. The Hyal vial should be stored unopened in a refrigerator [20]. If Hyal is stored at room temperature (25 °C), the stability is only guaranteed for 12 months [18]. The provider should follow the product guidelines for storage. The product should be injected immediately after preparation.

#### Hyal Dosage

##### Considerations

The Hyal dosage required depends on the indication (emergent vs nonemergent complication), location, volume, physical properties of the HAG to be dissolved, and patient factors [9,26]. The use of Hyal often involves a titration approach, where the practitioner assesses the response after each injection. Incremental adjustments of Hyal dosage are recommended—smaller doses and a gradual approach allow for fine-tuning, minimizing the risk of excessive filler degradation.

Vascular complications require larger doses than nonemergent (overcorrection, misplacement, and inflammatory reaction). Thinner skin (eg, lower lids and infraorbital areas) should be treated with lower Hyal doses. Larger filler volumes, larger particle size, higher concentrations of the filler, higher amount of cross-linking, and higher amount of G-prime contribute to increased durability of the filler requiring higher Hyal dosage for dissolution [9,26]. Also, monophasic (without distinct particles) HA formulations are more resistant to degradation than biphasic (particles suspended in gel) [26].

##### Physical Properties of HAGs

HA fillers have different physical properties that influence their degradation by Hyal in a time- and dose-dependent manner [21]. In an in vivo study using recombinant Hyal, Juvéderm Voluma required higher doses of Hyal than Restylane-L and Juvéderm Ultra for dissolution [11]. Therefore, Juvéderm Voluma may require repeat doses of Hyal for complete reversal.

A study by Rao et al [27] demonstrated Restylane (Galderma Laboratories) dissipated most and Belotero (Merz Pharmaceuticals) was most resistant to degradation. The authors showed that responses were similar for Vitrase and Hylenex, suggesting that these products can be used interchangeably. However, a subsequent study showed that Belotero was the fastest to degrade and Juvederm Voluma (Allergan) and Restylane Lyft were the slowest, with the authors concluding that a high concentration of HA, larger particle size, and increased cross-linking increase filler durability [9]. Jones et al [28] showed that Restylane and Prevelle (Mentor Corp) displayed greater sensitivity to ovine Hyal than Juvederm Ultra and contributed to the degradation resistance of Juvederm Ultra to higher HA content and level of cross-linking.

##### Drug Interactions

Drug interactions of Hyal should be considered. Salicylates, anti-inflammatories, cortisone, herbal meds, heparin, vitamin C, estrogens, and antihistamines make tissues resistant to Hyal [5,18]. One should consider a higher Hyal dosage or repeated injections in such cases. Therefore, having a thorough drug history before injecting Hyal is extremely important.

##### Dosage Recommendations for Nonemergent Complications

Regarding dosing, there are no accepted standardized guidelines. However, the rule of thumb for treating uncomplicated nodules is 5 iu Hyal for 0.1 mL HAG 20 mg/mL [16]. In the study by Zhang-Nunes et al [11] a cross-linked filler (Juvéderm Voluma, 20 mg/mL) required higher Hyal doses for dissolution, that is, more than 20 iu Hyal per 0.2 mL filler. In another study*,* in vivo degradation of cross-linked, highly cohesive HA fillers required 30 iu Hyal [29]. Woodward et al [30] recommended 30 iu to dissolve 0.1 mL. However, a study showed no statistical difference between using 20 or 40 iu Hyal in degrading 0.2 mL of various fillers (4-6 mg HA) [9]. Alam et al [10] showed that, although small Hyal doses (1.5-9 IU) can remove HA fillers, slightly higher doses often result in more rapid resolution.

Hyal dose for reversing overcorrection depends on the location and quantity of filler—in such cases, one may inject 15-30 iu in nasal or perioral areas, 3-4.5 in the periorbital area, 10-15 in the infraorbital area, and 1.5 in the lower [5]. However, even lower Hyal doses may be effective in reversing excessive augmentations. More resistant HAGs require higher Hyal doses of repetitive injections [21].

#### Skin Pretesting

As detailed in the section “Pitfalls” below, allergic reactions to Hyal are uncommon in aesthetics; they have been mainly reported in cases of peribulbar injection in the ophthalmology practice [31,32]. Therefore, no pretest is warranted in emergencies, such as vascular occlusions, as the risks of delaying the therapeutic intervention outweigh the potential benefit from pretesting [18,21]. However, bedside availability of epinephrine is required. Skin pretesting is considered optional when treating nonemergency complications of HAGs, such as overcorrection, superficial implantation, or inflammatory reactions. No pretesting is required for recombinant Hyal but may be considered for ovine, bovine, or compounded Hyals.

The testing consists of intradermal injection of 0.02-0.05 mL Hyal (to achieve a bleb of 5 mm) followed by observation for local wheal and flare within 5 minutes [21,24]. It is positive if such a reaction persists for 20-30 minutes. There is a lack of consistency regarding the optimal Hyal dose or concentration for pretesting. Doses 5-16 iu have been chosen [21,24], with the proponents of the higher doses indicating that lower doses may be unreliable since the drug causes an irritant reaction that could be misinterpreted as an allergy.

Before injecting Hyal, one should check for possible or conformed allergy to bee and wasp stings; such allergies pose a significant risk of cross-reactivity [24,33]. There are no standard precautions for using Hyal in patients allergic to bee and wasp stings [21]. In nonemergent filler complications, when a history of a large, localized reaction or anaphylaxis to bee or wasp stings exists, an intradermal test by an allergist is recommended. In emergent complications requiring Hyal in such a patient, the risks and benefits of not performing a skin pretest should be weighed [21].

#### Managing Filler Complications

##### Overview

This section reviews the elective use of Hyal for complications such as the Tyndall effect, noninflamed nodules resulting from overcorrection or misplacement of HA filler, inflammatory nodules, and allergic or immunogenic reactions to HA filler (Table 2). It also details the emergency use of Hyal in managing vascular occlusion to prevent tissue necrosis and blindness from periocular emboli. We discuss Hyal dosing for such complications and present our experience with Hyal treatments.

**Table 2:** Hyaluronidase dosage and considerations for treating complications of facial filler injections.

Aesthetic indication	Hyal dosage^a^	Hyal dosage (authors’ experience)	Considerations^b^
Tyndall effect	10-75 iu^c^ [2,34]	≤150 iu	Nature of HA^d^ filler (eg, cross-linked) Patient’s wish to maintain cosmetic benefit of filler injection
Noninflammatory nodules (overfilling or misplacement)	5-150 iu [21,25]	≤300 iu or more, depending on severity and filler type and volume	Nature and location of filler Volume of filler to be degraded
Asymmetry or contour irregularities	As above	≤225 iu	As above
Inflammatory nodules	500 iu every 48 hours to be administered after OAB^e^ have been tried for ≥2 weeks [39]; 30-300 iu combined with OAB [40]	Variable; often in conjunction with other treatments	Results of skin biopsy Results of microbiology testing (if nodule fluctuant or abscess) Nature of HA filler (eg, cross-linked)
Vascular occlusion	450-1500 iu total (high-dose protocol) [60] in up to 4 Hyal cycles; 35-50 iu under HFUS^f^ guidance (low dose protocol) [6]	300-1000 iu or more, depending on size of ischemic area	Nature of HA filler (eg, cross-linked) Size of ischemic area Embolus size Timing of intervention Patient factors (eg, scar in the area) HFUS imaging availability

^a^Multiple Hyal sessions are often required, and the provider may use incremental dose adjustments depending on the response.

^b^Considerations are crucial to decision-making and building an individualized approach to Hyal therapy.

^c^iu: international unit.

^d^HA: hyaluronic acid.

^e^OAB: oral antibiotics.

^f^HFUS: high-frequency ultrasound.

##### Tyndall Effect

When particulate HA fillers are inappropriately injected too superficially, a bluish discoloration (Tyndall effect) can result and may persist for a long time [1]. Treatment with 30-75 iu Hyal can be effective [34]—a smaller number (10-20 iu) may be used if a small amount of HA needs to be degraded [2]. This will often lead to complete resolution of the complication within 24 hours, although occasionally, a second Hyal treatment may be required [24]. The focus of the provider is on degrading the superficially placed filler. Also, Hyal dosage depends on whether the patient requests the filler to be completely removed or just eliminate the Tyndal effect [35]. The practitioner should follow up with the patient in 3-4 days to check whether additional Hyal is needed. Hyal may be used at any time and has been effective 63 months after the initial injection of HA [36].

##### Noninflamed Nodules

**Figure 1:**
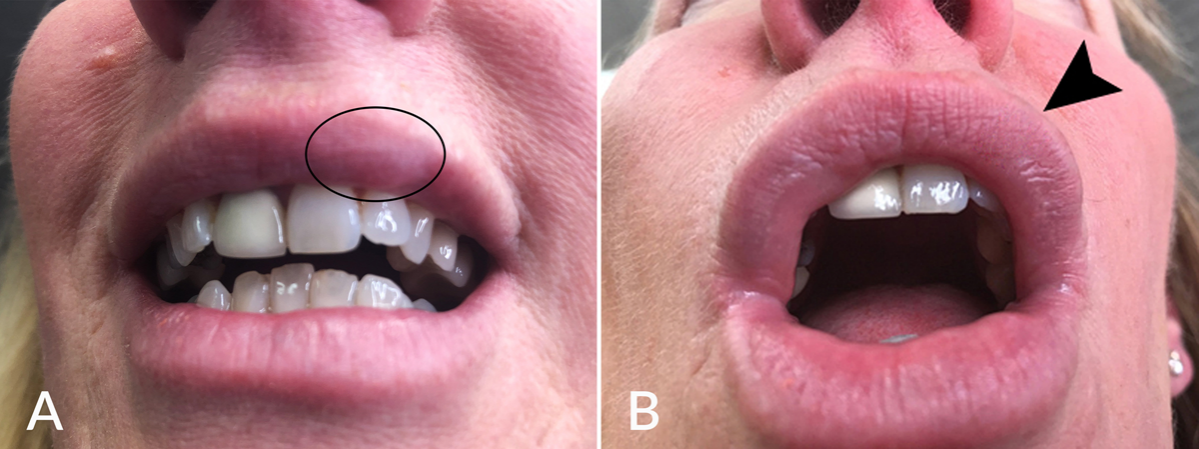
(A) Noninflamed nodule (encircled) developed after HA filler misplacement on the upper lip vermillion. (B) Lesion resolved (arrow) after injecting 15 iu recombinant human Hyal. HA: hyaluronic acid.

**Figure 2:**
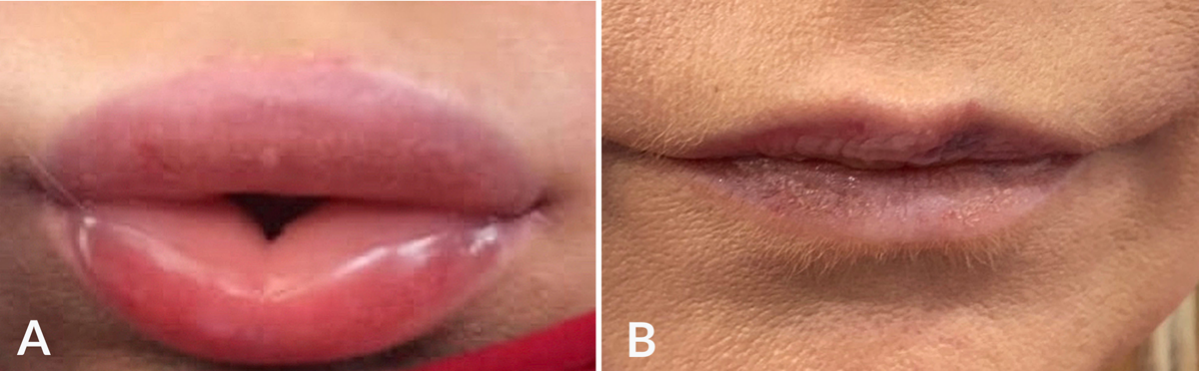
(A) Upper lip overfilling and edema that developed after HA filler overcorrection on the upper vermillion. (B) Complication resolved after injecting 150 iu recombinant human Hyal. As an allergic reaction was considered, intramuscular epinephrine (1:1000 solution) and 100 mg intravenous hydrocortisone therapy were also provided.

Noninflamed nodules are typically firm, feel rubbery, and tend to be painless. They are not usually associated with redness or significant discoloration. Their incidence is unknown. Noninflamed nodules result from overcorrection, filler misplacement, or migration (Figures 1 and 2). HFUS imaging is a first-line tool to identify the filler, assess its size, and exclude a soft tissue neoplasm (peer-reviewed by Kroumpouzos et al [1]). It may also help identify severe distant filler migration. Treatment of noninflamed nodules is warranted if painful, aesthetically bothersome, or associated with prolonged edema (ie, malar edema for >4 weeks). Hyal is delivered to the skin and subcutaneous tissue by directly infiltrating the visible or palpable HA depot [21]. Massage is recommended to mix the enzyme with HA and promote filler degradation. One may treat with a low dose, that is, 5-15 iu Hyal, and reassess in 1 week. However, higher doses (up to 150 iu) have been reported as effective [21,25]. As mentioned above, the volume and properties of the filler to be dissolved should be considered when deciding the dose to inject. A prospective trial included 8 participants who received 3 injections with 0.2 mL HA, and after 3-5 days each site was injected with 10, 20, or 30 iu Hyal. There were no differences among Hyal doses [8]. However, the study is limited by the small size and not including higher Hyal doses (ie, >50 iu).

The location of the filler should be considered as areas with thin skin, such as the eyelids, require low Hyal dosage (1.5-3 iu). Injecting a low dose helps prevent the loss of the HAG treatment effect. A retouch of another 1.5-3 iu 2-3 days later can be considered. Precautions to prevent ecchymosis should be taken when injecting the eyelids and infraorbital areas, especially as Hyal has been reported to spread the ecchymosis in these areas [37]. One should use a thin needle (30 G or thinner) and a single needle insertion point that helps minimize tissue trauma from the injection.

##### Undesired Aesthetic Outcomes

To prevent suboptimal aesthetic outcomes, the injector should consider patient characteristics, choose an appropriate filler for the area to be injected, avoid overfilling, and inject with a knowledge of anatomy. Overfilling can result in nodule formation and filler migration. Still, asymmetries, nodules, and other contour irregularities can occur even when patients are injected by experienced providers. Hyal is an appropriate therapy for such complications caused by HA fillers. The dosage approach is like that detailed for noninflamed nodules above. One should consider the amount of filler that needs to be degraded and titrate the Hyal dosage according to the response. These authors have used 150-300 iu Hyal for such complications (Table 2).

##### Inflammatory Nodules

Inflammatory nodules are often red and may feel warm to the touch. They can be associated with tenderness or pain. Inflammation may result from an immune response to the filler material, infection, or other complications [1]. Delayed-onset nodules (DONs) are usually inflammatory (ie, immune response to filler material), granulomatous (on histology), or related to infection or biofilm [1]. DON formation has a 0.5% incidence, a median time of onset of 4 months, and a median time to resolution of 6 weeks [38]. A subsequent retrospective study reported an incidence of 1% [39]. A skin biopsy and microbiologic testing should rule out granuloma formation and infection. A culture test of a draining or fluctuant lesion can aid in antibiotic selection. If an infection is suspected, oral antibiotic therapy should be administered, and the nodule should undergo incision and drainage if fluctuant. The American Society of Dermatologic Surgery recommended that noninflamed DONs without suspicion of infection might be treated initially with oral steroids for 1 to 2 weeks, rather than Hyal, should the retention of the aesthetic filler effect be desired [40]. The addition of antibiotics (doxycycline or minocycline) can be considered for anti-inflammatory and antimicrobial properties.

Regarding inflamed DONs, an expert panel recommended that high Hyal doses (ie, 500 iu every 48 hours until resolution) be administered after oral antibiotics have been tried for at least 2 weeks [41]. The panelists indicated that Hyal may break down the bacterial biofilm, thus facilitating the spread of infection; therefore, it should not be used as first-line therapy for inflammatory DONs. Another expert panel favored administering Hyal injection (30-300 iu) as first-line therapy with oral antibiotics [42]. Participating experts recommend a watchful approach of 48 hours to 2 weeks after starting antibiotic therapy, unless a more resistant HA (ie, Vycross) has been injected, in which case Hyal must be given as early as possible. Vycross technology has a 1% to 4% DON risk [40]. Highly cross-linked fillers may require higher doses and more sessions of Hyal for effective degradation due to their resilient nature. Early intervention with Hyal is preferred to prevent the development of more persistent complications. The above dose recommendations were made while also acknowledging that the Hyal dose depends on the size of the nodule, location (eg, tear troughs require a lower dose than midface), and filler properties [41,42]. The clinical practice supports injecting Hyal into the center of the nodule with a low gauge (18 or 21 G) needle to disrupt an encapsulated (filler) organization by allowing more penetrations [42]. Subsequent dissolution via Hyal with increasing dosages should be repeated after 2-3 weeks; however, Hyal injections should be limited to 2-3 cycles if there has been no response [42].

HFUS-guided injection can increase the likelihood of response of a nodule or granuloma to Hyal [16]. The inflamed nodule or granuloma has a “capsule” (ie, prominent chronic inflammatory and granulomatous reaction at the periphery); in such case, ultrasound can show in real time that the needle or cannula injecting the medication has penetrated the “capsule” before Hyal is injected [1].

Granuloma is a rare complication (0.01%-1%) of fillers and appears after a latent period, which can be several months to years postinjection [43,44]. Granulomas caused by HAs appear as cystic granulomas [45]. Encapsulation occurs at advanced stages, and histology shows palisaded granulomatous tissue mainly composed of giant cells and macrophages. Biofilm formation has been a suggested trigger [46]. Granulomas can be treated with Hyal dosed up to 150 iu [47]. Multiple Hyal sessions are often required. Granulomas with conspicuous fibrosis and abundant giant cells may not respond to Hyal. Still, most authors suggest using as first-line therapy high-concentration intralesional steroids, such as 20-40 mg/mL of triamcinolone or a combination of intralesional triamcinolone 10 mg/mL, 5-fluorouracil 50 mg/mL, and lidocaine [48]. Intralesional steroids interfere with the activities of fibroblasts, macrophages, giant cells, and collagen synthesis [45]. Intralesional steroids should be considered when inflammation is a significant component of the granulomatous reaction and 5-fluorouracil when there is excessive tissue growth associated with the granuloma. Treatment should be repeated every 3 to 4 weeks until resolution [48]. Surgical excision should be the last resort.

##### Allergic and Hypersensitivity Reactions

Most reactions to HA fillers are localized and manifest with edema, induration, and erythema at the injection site, pruritus, pain or tenderness, and eruption as early as a few days and as late as years after injection [49,50]. There have been no reports of type II or III reactions. Type I hypersensitivity reactions, such as localized angioedema, are uncommon as are type IV (delayed) reactions that are noted in less than 1% of cases [51]. Type IV reactions can manifest with painful erythematous nodules [50]. A delayed onset facial edema may be caused by type IV reaction and can develop several days to weeks after filler injection [52].

Type I reactions typically respond to oral antihistamines with or without intralesional or oral steroids [1,48,50]. Epinephrine should be administered in systemic reactions such as anaphylaxis or other severe cases. Providers should have an emergency kit containing epinephrine pens, oral steroids, and antihistamines in the treatment room [1]. Type IV reactions may not respond to antihistamines. Degradation of the filler depot with Hyal can be considered when an allergic or hypersensitivity reaction does not improve with a course of antihistamines or systemic corticosteroids. If the reaction is considered moderate or severe, oral corticosteroids should be taken before Hyal use to manage or prevent the potential initial worsening of symptoms due to increased antigens as the HA is broken down [52]. Hyal can be an alternative treatment for delayed facial edema as it does not carry the risks associated with prolonged systemic steroid treatment [52]; however, multiple treatment sessions are typically required, and Hyal can lead to at least partial loss of the filler treatment effect that may not be acceptable by the patient. COVID-19 vaccines have been reported to cause delayed reactions to HA fillers [53]. Two cases of COVID-19 vaccine-triggered delayed inflammatory reaction to HA filler were treated with Hyal [54,55].

##### Vascular Compromise and Skin Necrosis

**Figure 3:**
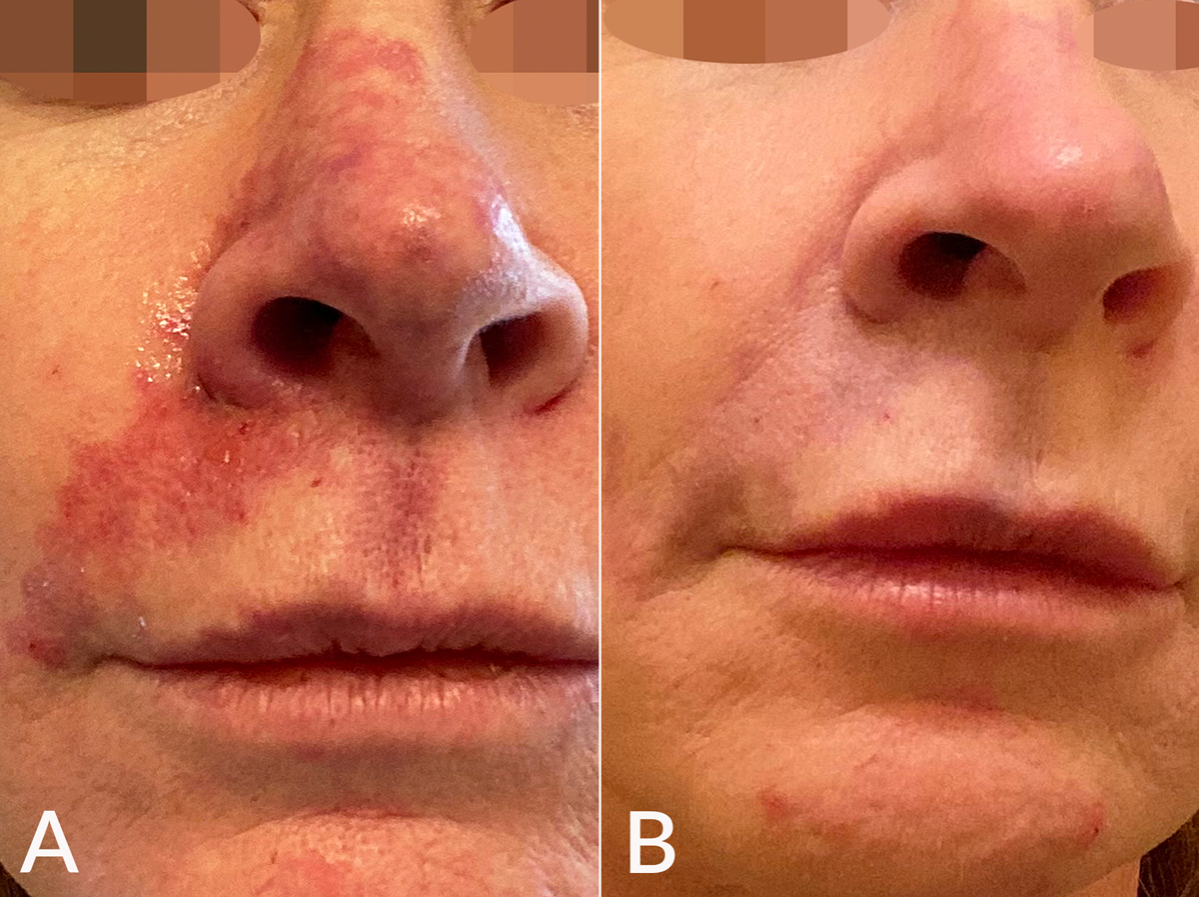
(A) Vascular compromise after embolization of the angular artery with HA injected in the nasolabial fold manifested with a livedoid pattern over the right nasolabial fold, lateral upper cutaneous lip, and nose. (B) Resolution of the complication is shown 2 days after treatment with 700 iu Hyal and vigorous massage.

The incidence of impending necrosis following dermal filler treatment was estimated at 0.001% (1 in 100,000 cases) in 2013 [56] and increased to 0.009% in 2020 [57]. Vascular occlusion associated with filler injection may be due to intravascular embolism, extravascular compression, and vascular spasm [1]. Pain is the earliest symptom, and coolness, blanching (immediate; may be transient), and livedo pattern are the earliest signs (Figure 3) [57]. A delayed capillary refill (normal, 1-2 seconds) is noted within minutes. A blue-gray appearance follows within tens of minutes to hours due to deoxygenated blood in the tissue. Skin breakdown is noted within days, and the following repair phase lasts days to weeks [57,58].

Vascular compromise requires immediate treatment. However, there is no consensus regarding the Hyal protocol in this complication [7]. Hyal should be administered as soon as possible, optimally within 4 hours [5]. The number of Hyal sessions depends on the severity of the complication and how quickly the intervention occurs. An animal study showed a significant reduction of the ischemic areas within 4 hours of Hyal treatment but no improvement after 24 hours [59]. In a series of patients with impending nasal skin necrosis related to the nose and nasolabial fold augmentation with HA fillers, full resolution of the complication occurred when Hyal treatment was administered within 2 days [60]. In a systematic review, Hyal failed to eliminate the large area of necrosis but played a moderate role in earlier recovery in limited necrosis [61].

A high-dose (total of 450-1500 iu in up to 4 Hyal cycles) pulsed Hyal protocol should be adopted [18,21,62]. Delorenzi suggested a simplified determination of Hyal dosage in the high-dose protocol considering the size of the ischemic area [62]. For a “single area” (eg, one-half of the upper lip) low-volume vascular event (by definition, <0.1 mL of filler embolus) Hyal dose should be about 450 iu; if a second area is affected, such as the nose, then the dose would be 900 iu. Hyal should be infiltrated diffusely into the ischemic tissues, including the vessel’s course. Perivascular Hyal will permeate vascular walls. Delorenzi showed ex vivo that cross-linked HA contained within the intact artery is susceptible to hydrolysis by Hyal found outside the vessel in its immediate surroundings [63]. Hyal injections should be followed by warm compresses and vigorous massage of the areas to improve drug diffusion and enhance blood flow. Then one should observe and reassess skin color and capillary refill after 60 minutes [62]; however, other authors recommend assessment every 15-20 minutes [24]. If vascular compromise persists, repeat Hyal treatment for up to 4 cycles should be administered [18,62]. Daily follow-up should occur, and more Hyal treatment performed until there is a satisfactory resolution. If treatment is completed within 72 hours of the onset of ischemia, success is possible [62].

An important study by Schelke et al [6] showed that when Hyal is injected under HFUS guidance, lower dosages (35-50 iu) than those in current “high dose” protocols (>500 iu) can be used. This is due to the higher accuracy of Hyal injections performed under HFUS guidance. Also, the study showed that a single Hyal injection yields a full resolution of the vascular complication compared to hourly injections over several hours in the current, high-dose protocol.

The patient should be kept under observation in the clinic for any adverse reactions—when anaphylaxis to Hyal occurs, it is usually within minutes, but there have been cases of delayed onset [18]. All patients should be warned about allergic or anaphylactic response symptoms and instructed to seek medical attention promptly.

##### Vision Loss

This is a rare but severe complication. A literature review by Beleznay et al [64] identified 146 cases in 2019. In recently reported cases, the nasal region (56.3%) was at the highest risk, and HA filler was the most common (81.3%) cause of this complication. Blindness due to periocular embolism of HA is instant and associated with excruciating ocular pain. The mechanism of action of blindness after filler injection is thought to involve intra-arterial injection of filler followed by subsequent retrograde embolization into the ophthalmic artery system [64]. The retinal circulation needs to be restored within 60 to 90 minutes if the retina is to survive. Blindness is an emergency; the patient should be transferred immediately to the nearest hospital ophthalmology department [65].

Currently, there is no evidence-based, accepted standard of care for treating visual compromise caused by filler [64]. Treatments that have been used vary widely and successful attempts are rare. If an HA filler was used, Hyal should be injected into the skin at the injection site and along the path of anastomosing arteries. Retrobulbar Hyal (RBH) injection (150-200 iu in 2-4 mL of diluent) into the inferolateral orbit should be considered by practitioners who have appropriate experience and competence while waiting for an ambulance [66]. A total of 3 cases experienced partial or complete vision recovery after treatment with RBH, although only 1 case directly attributed success to the RBH [64]. In that case, full vision restoration was achieved with Hyal (450 iu as retrobulbar injections and 300 iu to surround the supraorbital and infraorbital foramina) in a patient who received HA fillers in the midface [67]. RBH did not improve vision in other reports [68,69]. Zhu et al [68] failed to show any improvement in visual loss following 1500 to 3000 iu RBH in 4 patients. The authors indicated that Hyal is ineffective at recanalizing the retinal artery occlusion or improving the visual outcome after 4 hours of the onset of blindness.

However, other authors have challenged the RBH approach because Hyal did not demonstrate the ability to cross the dural sheath of the optic nerve and reach an occlusion of the central retinal artery [70,71]. In a cadaver model, Hyal could not cross the optic nerve’s dura into the space where it could bathe the central retinal artery [70]. Most importantly, hardly 5 mm of the ophthalmic artery is exposed in the orbit that is not covered with dura. An alternative approach was suggested, injecting into the supraorbital or supratrochlear artery. In the supraorbital method, Hyal is injected into the supraorbital artery in the supraorbital foramen [72]. The supraorbital approach is less invasive than the retrobulbar and can be effective in cases where the blood vessel blockage is closer to the skin’s surface. This technique has resulted in 2 cases of immediate vision recovery [72,73]. This approach requires no special skills compared to retrobulbar injections which are technically difficult procedures even for a competent ophthalmological surgeon. Still, other authors have challenged the feasibility and practicality of the supraorbital approach as the supraorbital and supratrochlear arteries are difficult to cannulate [64,74]. However, ultrasound guidance may facilitate this approach [75].

#### Limitations

Pitfalls include the loss of HAG treatment effect and adverse effects of Hyal such as allergic reactions. High Hyal doses can result in complete loss of the HAG effect. In a retrospective review of 20 patients with lower eyelid edema post-HA filler injection, Hyal 20-75 iu (injected 0.2-0.5 mL) per region was administered. All patients responded to treatment without recurrence. However, in 2 cases, all injected HA was degraded, resulting in a loss of treatment effect [76]. To prevent loss of the HAG effect, most authors recommend multiple treatment sessions with smaller Hyal doses in nonemergent filler complications, such as noninflammatory nodules, with reassessment after each session. The patient should be consulted regarding at least partial filler effect loss when Hyal is injected.

Adverse effects of Hyal injections are mainly local and include pruritus, burning sensation, swelling, erythema, ecchymosis limited to the injection site, spread of infection, and allergic reactions [1,24,77]. A total of 3 cases of ecchymosis away from the Hyal injection site in the infraorbital area were reported by this author who suggested that Hyal may facilitate the spread of ecchymosis on thin skin [37].

The overall allergy rates are low, reported 0.03%-0.13% with peribulbar injections [24]. immunoglobulin E-mediated type I hypersensitivity with the Hyal doses administered in aesthetic medicine is rare (incidence about 0.1%), but it is quoted high (33%) with large intravenous doses (>200,000 iu) [13,24]. Delayed hypersensitivity (type IV reaction) to Hyal has been rarely reported in aesthetic practice [77-79]. A case report described delayed hypersensitivity after Hyal treatment of granulomatous HA reaction [78]. In case of severe allergy caused by exogenous Hyal, autologous serum may be considered in nonacute cases requiring accelerated removal of HA filler [19].

This narrative literature review is limited by the sole inclusion of studies published in English available in PubMed and Google Scholar, which may have excluded studies unavailable in English or indexed in other databases. There is a limited number of controlled studies. Many studies included small sample sizes and reported descriptive outcomes. There is controversy regarding the most effective Hyal protocol for managing HA filler-associated vision loss.

#### Conclusions

Properly used Hyal can resolve nonemergent HA filler complications. The physical properties of the HA filler influence its degradation by Hyal and higher Hyal doses are required for HAGs resistant to degradation. Emergent complications such as vascular occlusion with impending skin necrosis should be treated promptly with high Hyal doses flushed into ischemic tissues. Hyal treatment of vision loss has met limited success, and the injection technique, retrobulbar versus supraorbital, remains controversial. More sufficiently powered controlled studies are needed. Hyal treatment has an acceptable safety profile, with allergic or hypersensitivity reactions uncommon in aesthetic practice.

## Abbreviations

DON: delayed onset nodule
HA: hyaluronic acid
HAG: hyaluronic acid gel
HFUS: high-frequency ultrasound
Hyal: hyaluronidase
iu: international units
RBH: retrobulbar Hyal
RCT: randomized controlled trial

## References

[1] Kroumpouzos G, Harris S, Bhargava S, Wortsman X (2023). Complications of fillers in the lips and perioral area: prevention, assessment, and management focusing on ultrasound guidance. J Plast Reconstr Aesthet Surg.

[2] Bhargava S, Arora G, Kroumpouzos G, Treacy P (2022). Perioral complications. Prevention and Management of Aesthetic Complications.

[3] Talei B (2019). Complications of injectables in the perioral region. Facial Plast Surg.

[4] Galadari H, Krompouzos G, Kassir M, Gupta M, Wollina U, Katsambas A, Lotti T, Jafferany M, Navarini AA, Berg RV, Grabbe S, Goldust M (2020). Complication of soft tissue fillers: prevention and management review. J Drugs Dermatol.

[5] Cavallini M, Gazzola R, Metalla M, Vaienti L (2013). The role of hyaluronidase in the treatment of complications from hyaluronic acid dermal fillers. Aesthet Surg J.

[6] Schelke LW, Velthuis P, Kadouch J, Swift A (2023). Early ultrasound for diagnosis and treatment of vascular adverse events with hyaluronic acid fillers. J Am Acad Dermatol.

[7] Borzabadi-Farahani A, Mosahebi A, Zargaran D (2022). A scoping review of hyaluronidase use in managing the complications of aesthetic interventions. Aesthetic Plast Surg.

[8] Vartanian AJ, Frankel AS, Rubin MG (2005). Injected hyaluronidase reduces restylane-mediated cutaneous augmentation. Arch Facial Plast Surg.

[9] Juhász MLW, Levin MK, Marmur ES (2017). The kinetics of reversible hyaluronic acid filler injection treated with hyaluronidase. Dermatol Surg.

[10] Alam M, Hughart R, Geisler A, Paghdal K, Maisel A, Weil A, West DP, Veledar E, Poon E (2018). Effectiveness of low doses of hyaluronidase to remove hyaluronic acid filler nodules: a randomized clinical trial. JAMA Dermatol.

[11] Zhang-Nunes S, Ryu C, Cahill K, Straka D, Nabavi C, Czyz C, Foster J (2021). Prospective in vivo evaluation of three different hyaluronic acid gels to varying doses of hyaluronidase with long-term follow-up. J Plast Reconstr Aesthet Surg.

[12] Buhren BA, Schrumpf H, Hoff NP, Bölke E, Hilton S, Gerber PA (2016). Hyaluronidase: from clinical applications to molecular and cellular mechanisms. Eur J Med Res.

[13] Jung H (2020). Hyaluronidase: an overview of its properties, applications, and side effects. Arch Plast Surg.

[14] Rzany B, Becker-Wegerich P, Bachmann F, Erdmann R, Wollina U (2009). Hyaluronidase in the correction of hyaluronic acid-based fillers: a review and a recommendation for use. J Cosmet Dermatol.

[15] Papakonstantinou E, Roth M, Karakiulakis G (2012). Hyaluronic acid: a key molecule in skin aging. Dermatoendocrinol.

[16] Quezada-Gaón N, Wortsman X (2016). Ultrasound-guided hyaluronidase injection in cosmetic complications. J Eur Acad Dermatol Venereol.

[17] Bailey SH, Fagien S, Rohrich RJ (2014). Changing role of hyaluronidase in plastic surgery. Plast Reconstr Surg.

[18] King M, Convery C, Davies E (2018). This month's guideline: the use of hyaluronidase in aesthetic practice (v2.4). J Clin Aesthet Dermatol.

[19] Weber GC, Buhren BA, Schrumpf H, Wohlrab J, Gerber PA, Labrou N (2019). Clinical applications of hyaluronidase. Advances in Experimental Medicine and Biology, Vol 1148: Therapeutic Enzymes: Function and Clinical Implications.

[20] HYLENEX recombinant (hyaluronidase human injection). Food and Drug Administration.

[21] Landau M (2015). Hyaluronidase caveats in treating filler complications. Dermatol Surg.

[22] Hyalase package leaflet. UK Government Web Archive: The National Archives.

[23] Pierre A, Levy PM (2007). Hyaluronidase offers an efficacious treatment for inaesthetic hyaluronic acid overcorrection. J Cosmet Dermatol.

[24] Murray G, Convery C, Walker L, Davies E (2021). Guideline for the safe use of hyaluronidase in aesthetic medicine, including modified high-dose protocol. J Clin Aesthet Dermatol.

[25] Brody HJ (2005). Use of hyaluronidase in the treatment of granulomatous hyaluronic acid reactions or unwanted hyaluronic acid misplacement. Dermatol Surg.

[26] Paap MK, Silkiss RZ (2020). The interaction between hyaluronidase and hyaluronic acid gel fillers—a review of the literature and comparative analysis. Plast Aesthet Res.

[27] Rao V, Chi S, Woodward J (2014). Reversing facial fillers: interactions between hyaluronidase and commercially available hyaluronic-acid based fillers. J Drugs Dermatol.

[28] Jones D, Tezel A, Borrel M (2010). In vitro resistance to degradation of hyaluronic acid dermal fillers by ovine testicular hyaluronidase. Dermatol Surg.

[29] Shumate GT, Chopra R, Jones D, Messina DJ, Hee CK (2018). In vivo degradation of crosslinked hyaluronic acid fillers by exogenous hyaluronidases. Dermatol Surg.

[30] Woodward J, Khan T, Martin J (2015). Facial filler complications. Facial Plast Surg Clin North Am.

[31] Lee A, Grummer SE, Kriegel D, Marmur E (2010). Hyaluronidase. Dermatol Surg.

[32] Delaere L, Zeyen T, Foets B, Van Calster J, Stalmans I (2009). Allergic reaction to hyaluronidase after retrobulbar anaesthesia: a case series and review. Int Ophthalmol.

[33] Keller EC, Kaminer MS, Dover JS (2014). Use of hyaluronidase in patients with bee allergy. Dermatol Surg.

[34] Urdiales-Gálvez F, Delgado NE, Figueiredo V, Lajo-Plaza JV, Mira M, Moreno A, Ortíz-Martí F, Del Rio-Reyes R, Romero-Álvarez N, Del Cueto SR, Segurado MA, Rebenaque CV (2018). Treatment of soft tissue filler complications: expert consensus recommendations. Aesthetic Plast Surg.

[35] King M (2016). Management of tyndall effect. J Clin Aesthet Dermatol.

[36] Soparkar CNS, Patrinely JR, Tschen J (2004). Erasing restylane. Ophthalmic Plast Reconstr Surg.

[37] Kroumpouzos G (2021). Extensive ecchymosis and edema associated with injection of human hyaluronidase in the periorbital area: a report of three cases. Dermatol Ther.

[38] Beleznay K, Carruthers JDA, Carruthers A, Mummert ME, Humphrey S (2015). Delayed-onset nodules secondary to a smooth cohesive 20 mg/mL hyaluronic acid filler: cause and management. Dermatol Surg.

[39] Sadeghpour M, Quatrano NA, Bonati LM, Arndt KA, Dover JS, Kaminer MS (2019). Delayed-onset nodules to differentially crosslinked hyaluronic acids: comparative incidence and risk assessment. Dermatol Surg.

[40] Jones DH, Fitzgerald R, Cox SE, Butterwick K, Murad MH, Humphrey S, Carruthers J, Dayan SH, Donofrio L, Solish N, Yee GJ, Alam M (2021). Preventing and treating adverse events of injectable fillers: evidence-based recommendations from the American Society for Dermatologic Surgery Multidisciplinary Task Force. Dermatol Surg.

[41] Philipp-Dormston WG, Goodman GJ, De Boulle K, Swift A, Delorenzi C, Jones D, Heydenrych I, De Almeida AT, Batniji RK (2020). Global approaches to the prevention and management of delayed-onset adverse reactions with hyaluronic acid-based fillers. Plast Reconstr Surg Glob Open.

[42] Artzi O, Cohen JL, Dover JS, Suwanchinda A, Pavicic T, Landau M, Goodman GJ, Ghannam S, Al Niaimi F, van Loghem JAJ, Goldie K, Sattler S, Cassuto D, Lim TS, Wanitphakdeedecha R, Verner I, Fischer TC, Bucay V, Sprecher E, Shalmon D (2020). Delayed inflammatory reactions to hyaluronic acid fillers: a literature review and proposed treatment algorithm. Clin Cosmet Investig Dermatol.

[43] Ramzi AA, Kassim M, George JV, Amin A (2015). Dental procedures: is it a risk factor for injectable dermal fillers?. J Maxillofac Oral Surg.

[44] Shahrabi-Farahani S, Lerman MA, Noonan V, Kabani S, Woo SB (2014). Granulomatous foreign body reaction to dermal cosmetic fillers with intraoral migration. Oral Surg Oral Med Oral Pathol Oral Radiol.

[45] Lee JM, Kim YJ (2015). Foreign body granulomas after the use of dermal fillers: pathophysiology, clinical appearance, histologic features, and treatment. Arch Plast Surg.

[46] Chiang YZ, Pierone G, Al-Niaimi F (2017). Dermal fillers: pathophysiology, prevention and treatment of complications. J Eur Acad Dermatol Venereol.

[47] Gupta A, Miller PJ (2019). Management of lip complications. Facial Plast Surg Clin North Am.

[48] Lemperle G, Duffy DM (2006). Treatment options for dermal filler complications. Aesthet Surg J.

[49] De Boulle K (2004). Management of complications after implantation of fillers. J Cosmet Dermatol.

[50] De Boulle K, Heydenrych I (2015). Patient factors influencing dermal filler complications: prevention, assessment, and treatment. Clin Cosmet Investig Dermatol.

[51] Chung KL, Convery C, Ejikeme I, Ghanem AM (2020). A systematic review of the literature of delayed inflammatory reactions after hyaluronic acid filler injection to estimate the incidence of delayed type hypersensitivity reaction. Aesthet Surg J.

[52] King M (2017). Management of edema. J Clin Aesthet Dermatol.

[53] Kroumpouzos G, Paroikaki ME, Yumeen S, Bhargava S, Mylonakis E (2022). Cutaneous complications of mRNA and AZD1222 COVID-19 vaccines: a worldwide review. Microorganisms.

[54] Michon A (2021). Hyaluronic acid soft tissue filler delayed inflammatory reaction following COVID-19 vaccination—a case report. J Cosmet Dermatol.

[55] Obagi S, Obagi Z, Altawaty Y, Obagi Z (2022). Treatment of delayed inflammatory response to hyaluronic acid soft tissue filler in a Pfizer-boosted Moderna-vaccinated individual with hyaluronidase. Surg Cosmet Dermatol.

[56] DeLorenzi C (2013). Complications of injectable fillers, part I. Aesthet Surg J.

[57] King M, Walker L, Convery C, Davies E (2020). Management of a vascular occlusion associated with cosmetic injections. J Clin Aesthet Dermatol.

[58] DeLorenzi C (2014). Complications of injectable fillers, part 2: vascular complications. Aesthet Surg J.

[59] Kim DW, Yoon ES, Ji YH, Park SH, Lee BI, Dhong ES (2011). Vascular complications of hyaluronic acid fillers and the role of hyaluronidase in management. J Plast Reconstr Aesthet Surg.

[60] Sun ZS, Zhu GZ, Wang HB, Xu X, Cai B, Zeng L, Yang JQ, Luo SK (2015). Clinical outcomes of impending nasal skin necrosis related to nose and nasolabial fold augmentation with hyaluronic acid fillers. Plast Reconstr Surg.

[61] Ors S (2020). The effect of hyaluronidase on depth of necrosis in hyaluronic acid filling-related skin complications. Aesthetic Plast Surg.

[62] DeLorenzi C (2017). New high dose pulsed hyaluronidase protocol for hyaluronic acid filler vascular adverse events. Aesthet Surg J.

[63] DeLorenzi C (2014). Transarterial degradation of hyaluronic acid filler by hyaluronidase. Dermatol Surg.

[64] Beleznay K, Carruthers JDA, Humphrey S, Carruthers A, Jones D (2019). Update on avoiding and treating blindness from fillers: a recent review of the world literature. Aesthet Surg J.

[65] Walker L, King M (2018). This month's guideline: visual loss secondary to cosmetic filler injection. J Clin Aesthet Dermatol.

[66] Carruthers JDA, Fagien S, Rohrich RJ, Weinkle S, Carruthers A (2014). Blindness caused by cosmetic filler injection: a review of cause and therapy. Plast Reconstr Surg.

[67] Chesnut C (2018). Restoration of visual loss with retrobulbar hyaluronidase injection after hyaluronic acid filler. Dermatol Surg.

[68] Zhu GZ, Sun ZS, Liao WX, Cai B, Chen CL, Zheng HH, Zeng L, Luo SK (2017). Efficacy of retrobulbar hyaluronidase injection for vision loss resulting from hyaluronic acid filler embolization. Aesthet Surg J.

[69] Hwang CJ, Mustak H, Gupta AA, Ramos RM, Goldberg RA, Duckwiler GR (2019). Role of retrobulbar hyaluronidase in filler-associated blindness: evaluation of fundus perfusion and electroretinogram readings in an animal model. Ophthalmic Plast Reconstr Surg.

[70] Paap MK, Milman T, Ugradar S, Silkiss RZ (2019). Assessing retrobulbar hyaluronidase as a treatment for filler-induced blindness in a cadaver model. Plast Reconstr Surg.

[71] Paap MK, Milman T, Ugradar S, Goldberg R, Silkiss RZ (2020). Examining the role of retrobulbar hyaluronidase in reversing filler-induced blindness: a systematic review. Ophthalmic Plast Reconstr Surg.

[72] Thanasarnaksorn W, Cotofana S, Rudolph C, Kraisak P, Chanasumon N, Suwanchinda A (2018). Severe vision loss caused by cosmetic filler augmentation: case series with review of cause and therapy. J Cosmet Dermatol.

[73] Goodman GJ, Roberts S, Callan P (2016). Experience and management of intravascular injection with facial fillers: results of a multinational survey of experienced injectors. Aesthetic Plast Surg.

[74] Fagien S, Carruthers J (2018). Commentary on restoration of visual loss with retrobulbar hyaluronidase injection after hyaluronic acid filler. Dermatol Surg.

[75] Schwenn OK, Wüstenberg EG, Konerding MA, Hattenbach LO (2005). Experimental percutaneous cannulation of the supraorbital arteries: implication for future therapy. Invest Ophthalmol Vis Sci.

[76] Hilton S, Schrumpf H, Buhren BA, Bölke E, Gerber PA (2014). Hyaluronidase injection for the treatment of eyelid edema: a retrospective analysis of 20 patients. Eur J Med Res.

[77] Kim MS, Youn S, Na CH, Shin BS (2015). Allergic reaction to hyaluronidase use after hyaluronic acid filler injection. J Cosmet Laser Ther.

[78] Wu L, Liu X, Jian X, Wu X, Xu N, Dou X, Yu B (2018). Delayed allergic hypersensitivity to hyaluronidase during the treatment of granulomatous hyaluronic acid reactions. J Cosmet Dermatol.

[79] Ebo DG, Goossens S, Opsomer F, Bridts CH, Stevens WJ (2005). Flow-assisted diagnosis of anaphylaxis to hyaluronidase. Allergy.

